# CT Scan of Thirteen Natural Mummies Dating Back to the XVI-XVIII Centuries: An Emerging Tool to Investigate Living Conditions and Diseases in History

**DOI:** 10.1371/journal.pone.0154349

**Published:** 2016-06-29

**Authors:** Enrico Petrella, Sara Piciucchi, Francesco Feletti, Domenico Barone, Antonella Piraccini, Caterina Minghetti, Giorgio Gruppioni, Venerino Poletti, Mauro Bertocco, Mirko Traversari

**Affiliations:** 1 Department of Radiology, Azienda USL Romagna, Ospedale G.B. Morgagni, Forlì, FC, Italy; 2 Department of Radiology; Santa Maria delle Croci Hospital, Ravenna, RA, Italy; 3 Radiology Unit; IRCCS Istituto Scientifico Romagnolo Per lo Studio e Cura dei Tumori (IRST), Meldola, FC, Italy; 4 Department of History and Methods for Conservation of Cultural Heritage of University of Bologna, BO, Italy; 5 Department of Diseases of the Thorax; Azienda USL Romagna, Ospedale GB Morgagni, Forlì, FC, Italy; 6 Department of Respiratory Diseases & Allergology, University Hospital, Aarhus, Denmark; University of Oulu, FINLAND

## Abstract

**Objectives:**

To correlate the radiologic findings detected with computed tomography scan with anthropological data in 13 naturally mummified bodies discovered during works of recovery of an ancient church in a crypt in Roccapelago, in the Italian Apennines.

**Methods:**

From a group of about sixty not-intentionally mummified bodies, thirteen were selected to be investigated with volumetric computed tomography (CT). Once CT scan was performed, axial images were processed to gather MPR and Volume Rendering reconstructions. Elaborations of these images provided anthropometric measurements and a non-invasive analysis of the residual anatomical structures. For each body the grade of preservation and the eventual pathological changes were recorded. Furthermore, in order to identify nutritional and occupational markers, radiologic signs of bone tropism and degenerative changes were analysed and graded.

**Results:**

Mummies included seven females and six males, with an estimated age ranging from 20 to 60 years. The first relevant finding identified was a general low grade of preservation, due to the lack of anatomic tissues different from bones, tendons and dehydrated skin. The low grade of preservation was related to the natural process of mummification. Analysing bone degenerative changes on CT scan, the majority of the bodies had significant occupational markers consisting of arthritis in the spine, lower limbs and shoulders even in young age. Few were the pathological findings identified. Among these, the most relevant included a severe bilateral congenital hip dysplasia and a wide osteolytic lesion involving left orbit and petrous bone that was likely the cause of death.

**Conclusions:**

Although the low grade of preservation of these mummies, the multidisciplinary approach of anthropologists and radiologists allowed several important advances in knowledge for the epidemiology of Roccapelago. First of all, a profile of living conditions was delineated. It included occupational and nutritional conditions. Moreover, identification of some causes of death and, most importantly the definition of general living conditions.

## Introduction

In the course of history, mummification represented the artificial procedure aimed to preserve body integrity of eminent men and women. The intentional mummification process was actually, diffuse among some relevant people of the Church or, before, in ancient Egypt among Pharaons. However, a rare possibility of non-intentional, natural process of mummification, primed by environmental conditions exists and can represent a huge source of investigation for paleoanthropological, paleopathological and paleoradiological research [1–2–3–4–5]. Radiology rose its interest on study of mummified bodies and paleoradiological studies guaranteed more and more accurate and non-invasive evaluation of anatomical structures and physical integrity [3–4–5–6–7–8].

The first publication in paleoradiology is dated in 1896, just one year after the discovery of X rays. In that occasion actually, a radiograph investigated the content of an Egyptian sarcophagus initially believed to belong to a person. X rays revealed instead that the body inside the sarcophagus belonged to a huge bird, probably an ibis that was mummified [9].

Since that time, radiographic examinations were more and more employed to study mummies thanks to the advantage of maintaining a perfect integrity of the bodies if compared with traditional methods of autopsy. In contrast with a significant amount of radiologic reports on engineered mummies, actually few papers describe the morphologic and radiologic appearance of naturally mummified bodies [10].

In the present study, we describe the exceptional event of a natural partial mummification or skeletonization of numerous subjects belonging to a mountain community of the North-West of Italy. Out of this population we scanned thirteen mummies with multislice computed tomography (CT).

The aim of this article is to investigate whether CT and post-processing techniques are useful screening tests to evaluate the status of preservation of bony and non-bony tissues. Moreover we investigated if the eventual diseases or causes of death may be inferred.

Secondly, we aimed our attention at the living conditions evaluating occupational and nutritional markers on CT scan.

Finally we tried to compare data obtained in our sample with epidemiologic data coming from the ancient records of Roccapelago (Parish Records).

## Materials and Methods

Currently the sample analyzed mummies is publicly deposited and visible at the public museum “Museo delle Mummie di Roccapelago”, built inside the San Paul’s Conversion Church of Roccapelago in Pievepelago [11]. Superintendence to Archaeological properties of Emilia-Romagna, with licenses with protocol numbers 9354 and 9359 pos. B15, 34.31.03/10 dated July 16, 2012, has provided permissions for anthropological and radiological study

### Discovery of Mummies

The discovery of our sample dates back to October 2009, the period of the restoration of Saint Paul’s Conversion Church of Roccapelago in Pievepelago. Roccapelago is a mountain site in the Apennines, close to Modena in the North-West of Italy.In the course of the restoration, many archeological excavations with remains of a Medieval village were discovered and a pre-existing church with multiple burials in the crypt of the church [12]. The crypt was used as a cemetery by the small community from the 16^th^ century until the Napoleonic law banning burials inside churches in the late 18th century.

Overall burials consisted of 281 bodies, including infants, children and adults. Among these buried bodies, about sixty showed signs of mummification. This mummification process mostly involved some parts of various bodies, because the other parts resulted as skeletonized or, sometimes, missing.

This process resulted from a natural mummification process, not engineered as traditionally happened for social groups like monks or members of eminent families.

It was likely induced by the mountain climate and by features of the crypt which had been built in a porous rock.

**Fig 1:**
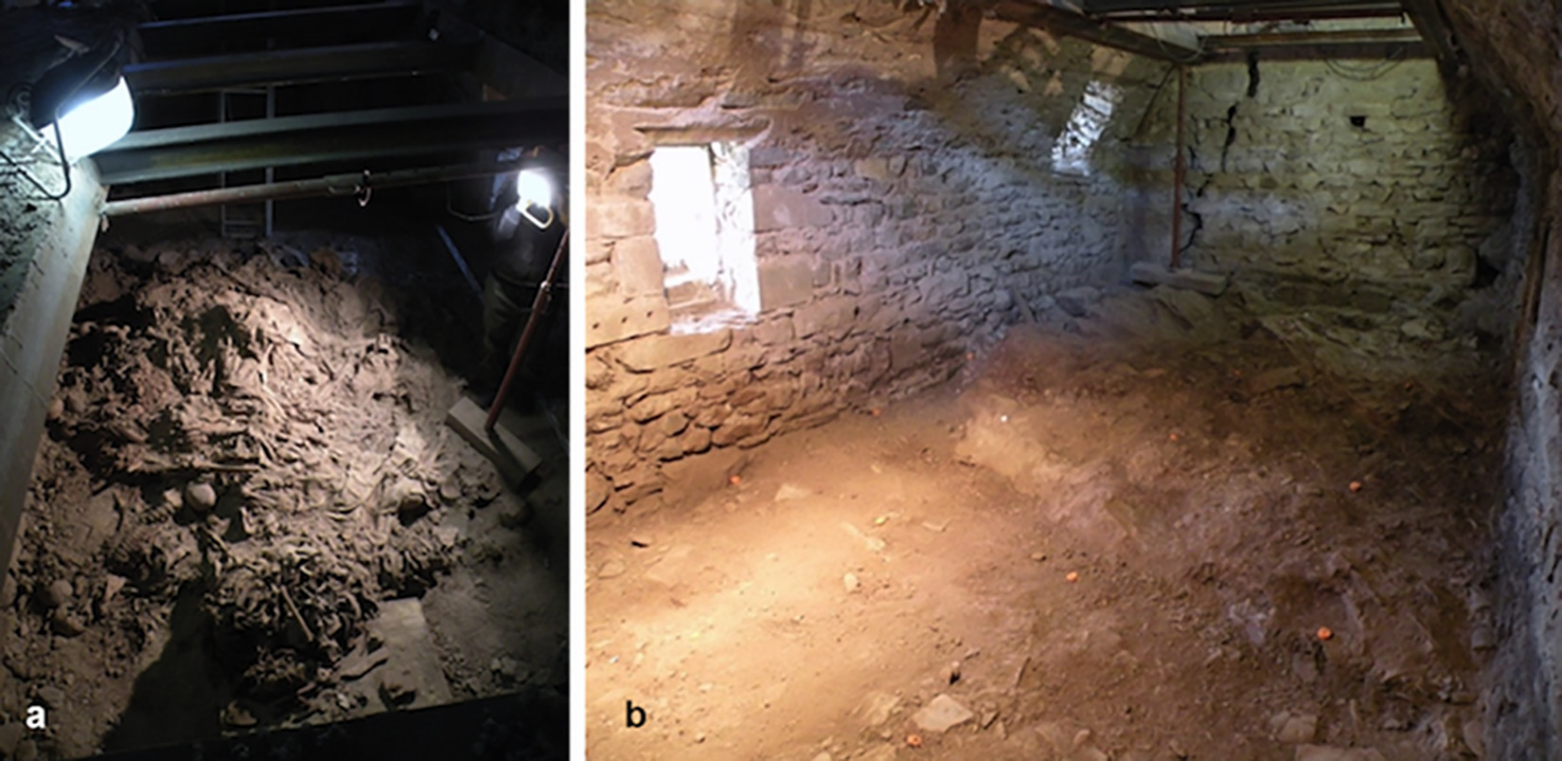
Bodies amassed in a pyramidal pile, at the moment of discovery in the crypt (a). Empty crypt after removing mummies. To note lateral window and floor in pure rock (b).

Moreover, the crypt had some little lateral windows that guaranteed the constant aeration (Fig 1) and the maintenance of the mummification.

From XVI century to Napoleonic laws in XVIII century, the crypt represented the rudimental cemetery of the village and dead bodies were lowered through a trap door in the ceiling (Fig 1A and 1B).

In the course of the centuries, dead bodies, each of them closed in an envelope made of jute, formed a pile with a pyramidal shape.

The amount of bodies discovered inside the crypt were about 281. A significant number of individuals, especially those coming from the most recent units (SU 23), preserved their anatomic connections and appeared only partially skeletonized, conserving substantial parts of mummified soft tissue.

### Musealization

After the discovery, each body was placed on a rigid support and transferred to the laboratory of Anthropology in Ravenna, at the Department of Cultural Heritage of University of Bologna. The following steps included: the immediate musealisation and creation of a biological archive of significant cases. The musealisation was carried out in compliance with the indications in the “Culturally Sensitive Material” code under art. 2.5, 3.7 and 4.3 of the “ICOM ethical code” [13].

### Anthropological evaluation of Mummies

All the sixty bodies were firstly examined by anthropologists, who tried to find out the gender and to estimate the age.

The hip-bone sexual characteristics were considered to estimate the gender using the Probabilistic Sex Diagnosis (DSP) [14].

The estimation of age-at-death, whenever possible, combining the analysis of the pubic symphysis [15–16], the iliac sacropelvic surface [17], and of the dentition [18]. Indeed, individuals were classified among five anthropological age classes (20–29 y., 30–49 y., 40–49 y.>50 y., >60 y.).

Successively anthropological evaluation considered occupational markers according to the analysis of biomechanical stress indicators using 3D virtual models of bones.[12].

Out of the 60 mummified bodies, thirteen were selected for radiologic investigation with the inclusion criteria of the best preservation and the highest percentage of mummified tissue.

Finally the selected sample of thirteen mummies were examined by multislice CT scan.

### CT evaluation of the mummies

**Fig 2:**
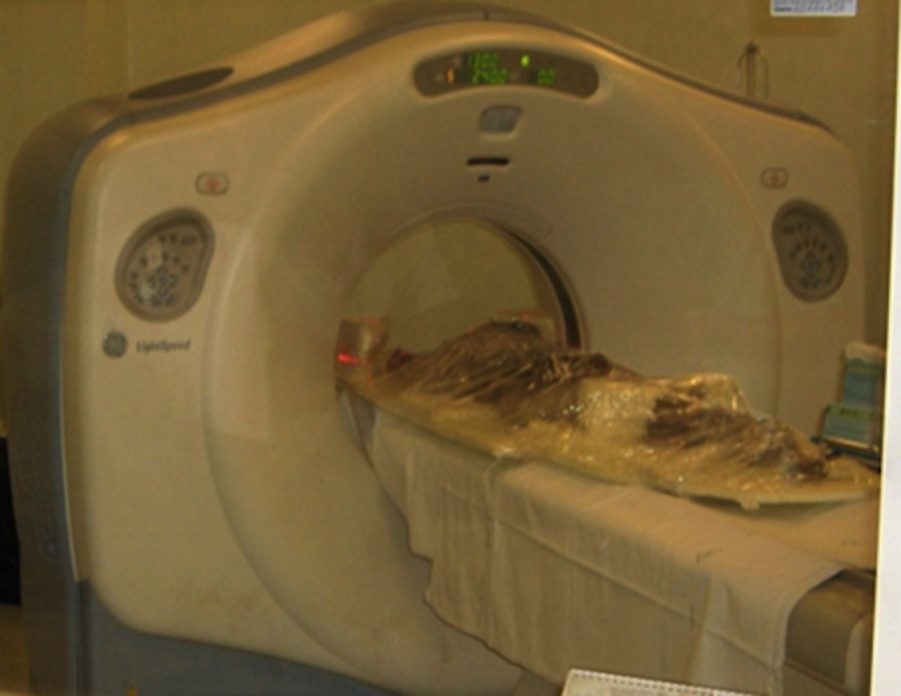
Mummies sited on rigid support and examined with CT scan.

Six subjects underwent CT scan at the department of Radiology of the GB Morgagni Hospital in Forlì and seven mummies were studied at the Radiology Department of Santa Maria delle Croci Hospital in Ravenna (Fig 2).

Tomographic studies consisted of a single volumetric scan for each mummy (Ravenna: 64 slices Brilliance, Philips Medical System, Eindhoven-The Netherlands; Forlì: CT scanner 16 slices Lightspeed General Electric Systems Milwaukee- WI).

Parameters of acquisition at the hospital of Forlì included: 1.25 slice thickness, with an interval of reconstruction of 0.7 mm; 120 kV and 140–300 mA.

Parameters of acquisition at the hospital of Ravenna included: slice thickness 1mm, interval of reconstruction 1mm, 80KV and 100–200 mA. Axial images were finally processed on a separate common workstation Philips Brilliance Portal Workspace 3D, in order to obtain multiplanar (MPR) and volumetric 3D reconstructions.

Post-processing CT study consisted of four steps for each case as described by Cesarani et Al. Egyptian Human Mummies [1].

#### The four-steps CT analysis of the mummies

Each mummified body was archived in the PACS system of each hospital using the same numeration system applied by anthropologists: “L” for the layer (SU) in which the mummy was discovered, “ENV” for the envelope in which was replaced and “IND N” as an abbreviation of individual numeration of each subject.

In the first step, axial images were analyzed through the different windows of visualization (bone and soft tissue filters) to identify residual anatomical structures with different densities. Coronal and sagittal MPR reconstructions were also performed to better analyse the “content” of the mummy.

In a second step, 3D reconstructions allow to obtain views of the overall external aspects of the mummies including residual clothes.

In the third step, Manual or semi-automated techniques remove clothes, bandages, and superficial fragments from the ground [7].

In the third step, the anatomic residual tissues were studied to assess preservation and integrity.

Preservation is represented by the amount of tissue (digestive, soft tissue, cartilage) relievable in each body.

Anatomic integrity represents the amount of anatomical segments present in each mummy.

In the fourth step, there is the attempt of inferring anthropometric data and epidemiologic information such as occupational and nutritional markers.

**Fig 3:**
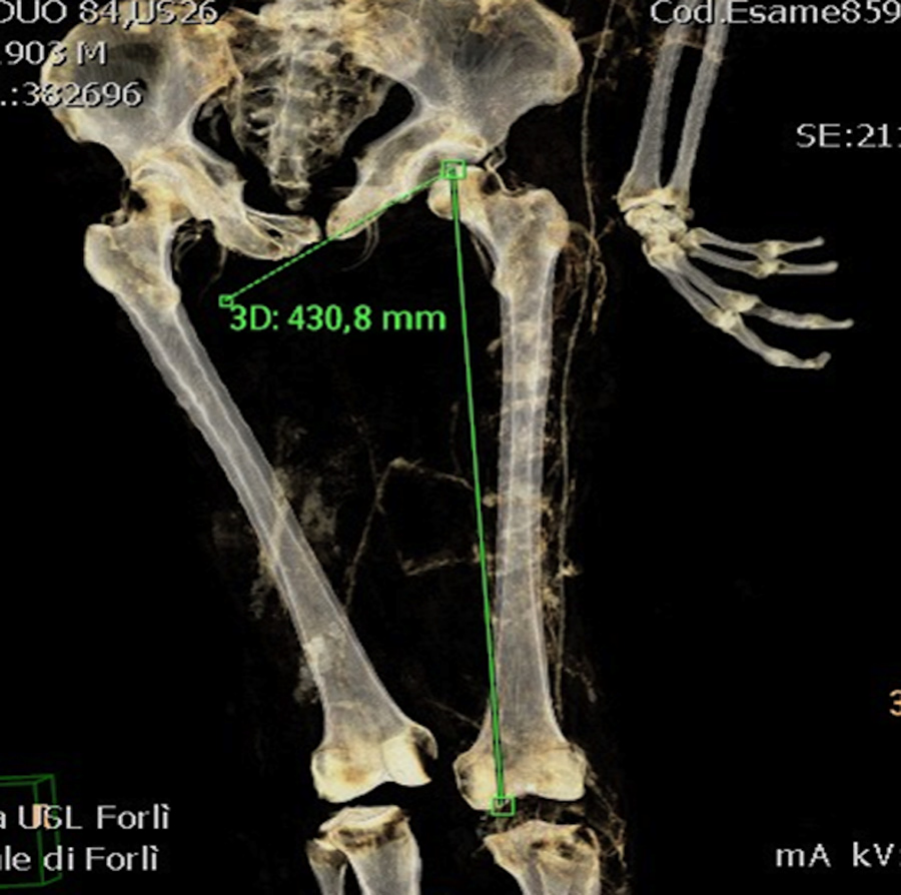
Measurement of left femur for anthropometric calculation of height.

With regards to anthropometry, measurements of the femoral length allowed an estimation of the height of each individual (Fig 3).

Through the identification of chronic degenerative changes such as arthritis in specific sites, we also obtained information about occupational activity. Specific degenerative changes such as spondyloarthritis and degenerative changes of shoulders, should be considered as “occupational markers”, particularly if present in a relatively young age, [12].

On the other hand, nutrition has been investigated through the mineralization grade of bones, looking for Looser lines. Looser lines represent wide transverse lucencies traversing part way through a bone. They are usually associated with osteomalacia and rickets [19]. Both nutritional and occupational markers allow us to infer the quality of life of an ancient community.

In the fourth step, we also analysed eventual pathologic changes such as neoplastic or chronic inflammatory lesions.

## Results

### CT analysis of Anatomical integrity

Following the modality of burying, eight mummies were set down in a supine, three in prone and two in lateral position.

Most bodies showed an acceptable anatomical integrity, except for three cases in which only few fragments were preserved. These three individuals showed only head and cervical spine in one case, pelvis and legs in the second and lumbar spine, part of the pelvis and legs in the third case.

A complete anatomical integrity was radiologically assessed only in two cases (L 23 ENV 60 IND 50 and L23 ENV 90 IND 56).

In the remaining cases, six mummies were acephalous (IND 12, 48, 57, 59, 84 and 85). Out of the six acephalous mummies, two mummies had their heads sited adjacent to the lower limbs (IND 53, 55).Three cases had only some anatomical fragments, particularly: head and cervical spine in one case (IND 55); part of the legs and of the pelvis in two cases (IND 12 and 48).

#### CT analysis of Preservation

With regards to internal organs, each mummy with a head showed residual meninges characterized by amorphous tissues associated with dehydrated encephalic tissue.

Regarding the grade of preservation of anatomical tissues, the best preservation was documented for meningeal membranes that were identifiable in all the heads and in most of the vertebral spines.

Tendons of hands and feet were also present in the majority of the cases.

Part of the skin of the trunk and the legs was partially present.

Soft tissues of the legs and torso looked like a shapeless mass without any anatomical identity.

**Fig 4:**
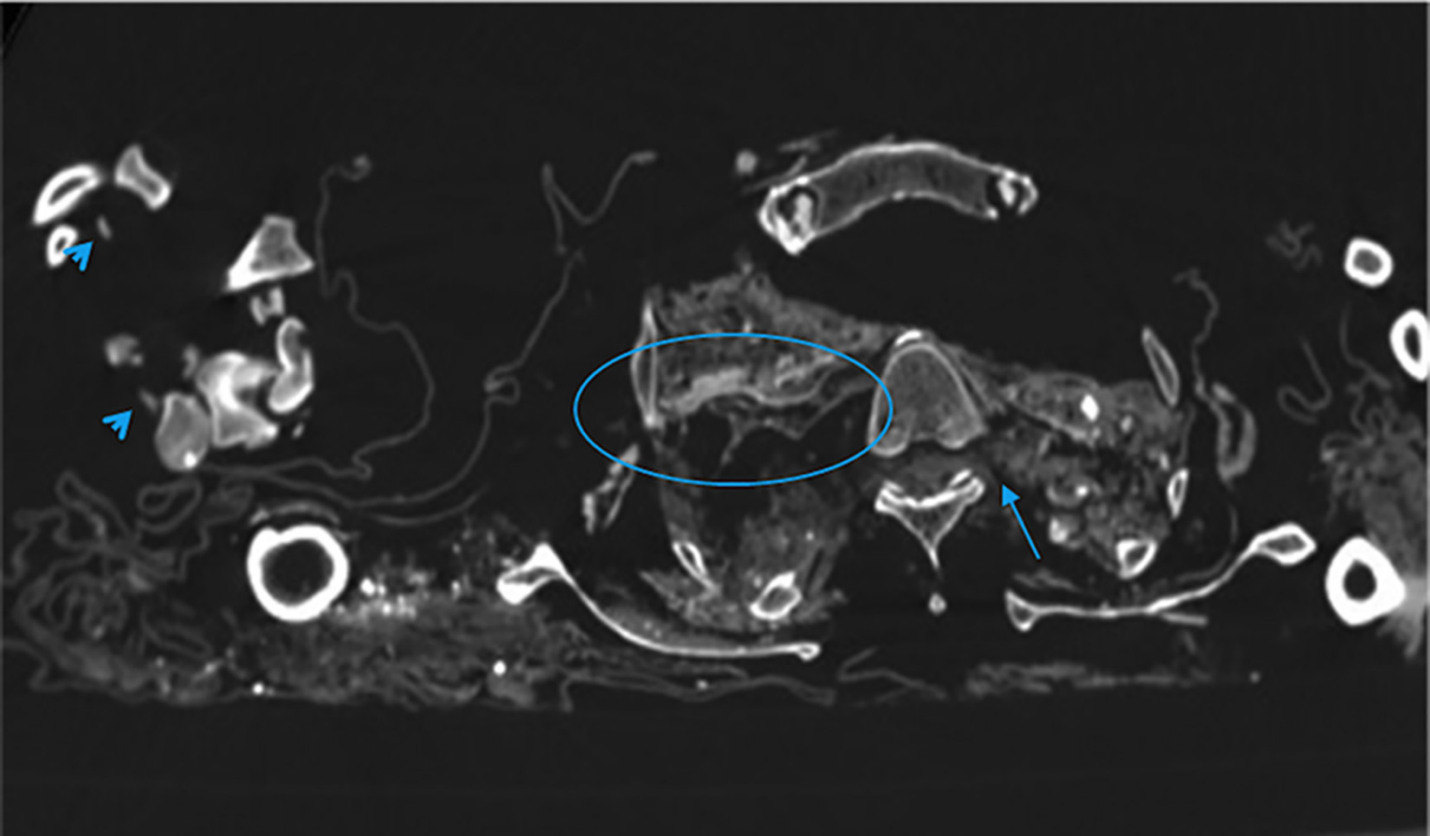
Soft tissues of the trunk without a clear anatomical identity. The tubular structure on the right side of the vertebral body may be residual of mediastinal organ, such as trachea (white circle). Residual of spinal medulla with left radicular emergence (arrow). Flexor tendons of right hand (arrow-heads). Multiple hyper attenuated inclusions, maybe related to little rocks, can be observed in axial scans.

These kinds of soft tissues were present also in the thorax and the abdomen close to several hyperattenuated enclosed elements likely due to debris and stones (Fig 4).

In one case we identified a tubular structure in the thorax that was likely a residual of trachea (Fig 4).

According to the recent report of Panzer et Al.[20] introducing the CT scoring for a quantitative grading of preservation of soft tissues and organs system, we calculated the score for each mummy.

Score of preservation of our sample was significantly low. Particularly the soft tissue score ranged from 8 to 58 and the organ system score ranged from 0 to 3 (Table 1).

**Table 1:** General features of the sample with estimated age, gender, anatomical integrity.

Mummy ID	Gender	Estimate age	Anatomical integrity(segments present)	Preservation Score	Femur	Height
**L 23 ENV 20 IND 12**	F	40–49	Legs—pelvis	•Soft tissue: 20 •Organs: 0	R 42,6 L, 27	159
**L 23 ENV 41 IND 48**	M	>60	Legs—pelvis	•Soft tissue: 12 •Organs: 0	R 44,4 L 43,4	168,5
**L 23 ENV 43 IND 49**	F	30–49	Head-trunk-Upper legs- femurs	•Soft tissue: 14 •Organs: 3	R 40,0 L 40,5	152
**L 23 ENV 60 IND 50**	F	20–29	Complete skeleton	•Soft tissue: 34 •Organs: 3	R 43,0 L 42,9	160
**L 23 ENV 87 IND 53**	F	40–49	Head- trunk- lower limbs-left upper leg	•Soft tissue: 24 •Organs: 1	R 42,3 L 42,5	159
**L 23 ENV 88 IND 54**	F	30–49	Head-Spine-upper legs-Right hip-right lower leg	•Soft tissue: 30 •Organs: 1	R 43	160
**L 23 ENV 89 IND 55**	M	>50	Trunk- upper and lower limbs—Missing feet	•Soft tissue: 20 •Organs: 1	R 42,6 L 42,8	165
**L 23 ENV 90 IND 56**	M	>50	Complete skeleton	•Soft tissue: 58 •Organs: 3	R 42,5 L,3	164
**L 23 ENV 91 IND 57**	F	>60	Upper and lower limbs trunk- missing one foot	•Soft tissue: 20 •Organs: 0	R 40 L 41,2	152
**L23 ENV 59 IND 59**	M	>50	Upper and lower limbs trunk- missing feet	•Soft tissue: 20 •Organs: 0	L 42,4	163
**L 26 ENV 92 IND 84**	F	>50	Upper and lower limbs trunk	•Soft tissue: 26 •Organs: 0	R 43,2 L43,1	160
**L 26 ENV 93 IND 85**	M	>50	Upper and lower limbs trunk	•Soft tissue: 26 •Organs: 0	R47,4 L 47,3	175
**L 26 IND 55**	M		Head cervical spine	•Soft tissue: 8 •Organs: 1	**——————**	——————

Length of both femurs or of the residual femur is reported with the consequent calculation of the height.

### Gender and age estimation

According the anthropologic analysis (that included both direct analysis on the body and on funerary trousseau) regarding gender and age, in our sample, seven subjects were females and five were males [14, 15–18].

In the specific case in which only head and cervical spine was available, we were not able to perform an estimation.

With regards to the age estimation, one subject was estimated to be about 20–29 years old, two mummies were 30–49 years old, one 40–49 years old, five were older than fifty and two older than sixty. Gender, estimated age, anatomical integrity, estimated height of each subject according to the anthropometric measurements of Bass [21], measuring length of femur are summarized in Table 1 (Fig 3)

### Pathological Changes

In the last step of analysis, we looked for pathological changes, including tumors, congenital diseases and inflammatory changes as summarized in Table 2.

**Table 2:** Cases of congenital diseases, tumours and inflammatory diseases.

Mummy ID	Tumours	Inflammatory diseases	Congenital diseases
**L 23 ENV 20 IND 12**			Bilateral hip dysplasia
**L 23 ENV 43 IND 49**	Cystic lesion left femur		
**L 26 ENV 92 IND 84**	Vertebral hemangioma		“En-block” vertebra
**L 26 ENV 93 IND 85**	Vertebral hemangioma		
**L 26 IND 55**		Osteomyelitis at basis of skull and left temporal region	

Degenerative changes of spine, of upper and lower limbs are reported according to a grading of disease published by Kellegren and Lawrence [22] (Table 3).

**Table 3:** Cases of chronic degenerative changes of the spine and of legs.

Mummy ID	Gender	Estimate age	Degenerative changes spine	Degenerative changes legs	Severity osteoarthitis
**L 23 ENV 20 IND 12**	F	40–49		Hips- knees	**4**
**L 23 ENV 41 IND 48**	M	>60	•Spondiloarthritis •Schmorl hernias		**4**
**L 23 ENV 43 IND 49**	F	30–49	•Spondiloarthritis •Schmorl hernias	Hips	**3**
**L 23 ENV 60 IND 50**	F	20–29			
**L 23 ENV 87 IND 53**	F	40–49			
**L 23 ENV 88 IND 54**	F	30–49			
**L 23 ENV 89 IND 55**	M	>50			
**L 23 ENV 90 IND 56**	M	>50	Spondiloarthritis		**2**
**L 23 ENV 91 IND 57**	F	>60		Distal epiphysis of tibia	**1**
**L23 ENV 59 IND 59**	M	>50	Spondiloarthritis	Hip, knee, humeral heads	**3**
**L 26 ENV 92 IND 84**	F	>50			
**L 26 ENV 93 IND 85**	M	>50	•Spondiloarthritis •Schmorl hernias		**3**
**L 26 IND 55**	M				

In the last column the grading of degenerative changes according to the Kellegren and Lawrence scale (20) is reported.

Seven subjects showed signs of osteoarthritis with a grading ranging from 1 to 4, and with a mean value of 2.9 according to the above cited grading [22].

**Fig 5:**
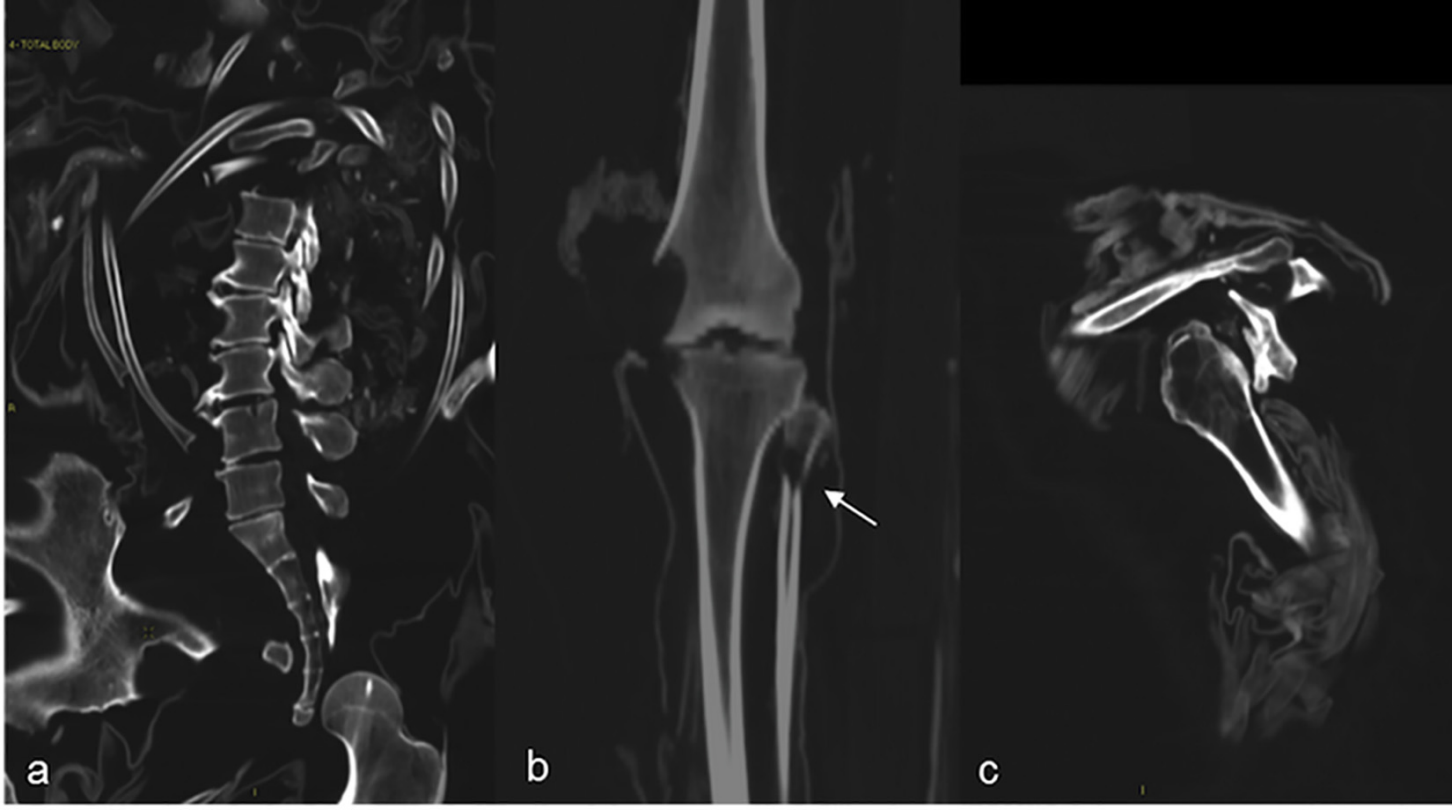
Degenerative signs of the lumbar spine, with moderate osteophytosis (a). Degenerative signs of knee with sclerosis of articular layers and osteophytosis. Fracture of proximal metaphysis of fibula without any bone reparative sign, suggestive of post-mortem fracture (arrow) (b). Sclerosis of the glena and irregularity of greater tuberosity (c).

We identified four subjects with initial degenerative changes such as spondyloarthritis in four cases (IND 59, 85, 48 and 49) three of whom with several Schmorl hernias (IND 85, 48 and 49) (Fig 5A).

The subject IND 59 presented signs of moderate arthritis to hip, knee (Fig 5B) and to both humeral heads (Fig 5C). Only on the left side of this subject significant signs of calcified tendinopathy were identified likely due to excessive use in a left-handed person.

Subject IND 49 presented bilateral arthritis of the hip as well. Subject IND 57 presented signs of sclerosis of the spongious bone at the distal epiphysis of both tibias, likely due to bone remodeling related to overload.

Two subjects (IND 84 and 85) showed vertebral hemangiomas and IND 84 showed a “en block-vertebra” T11-T12.In three individuals we also observed frankly pathological changes.

**Fig 6:**
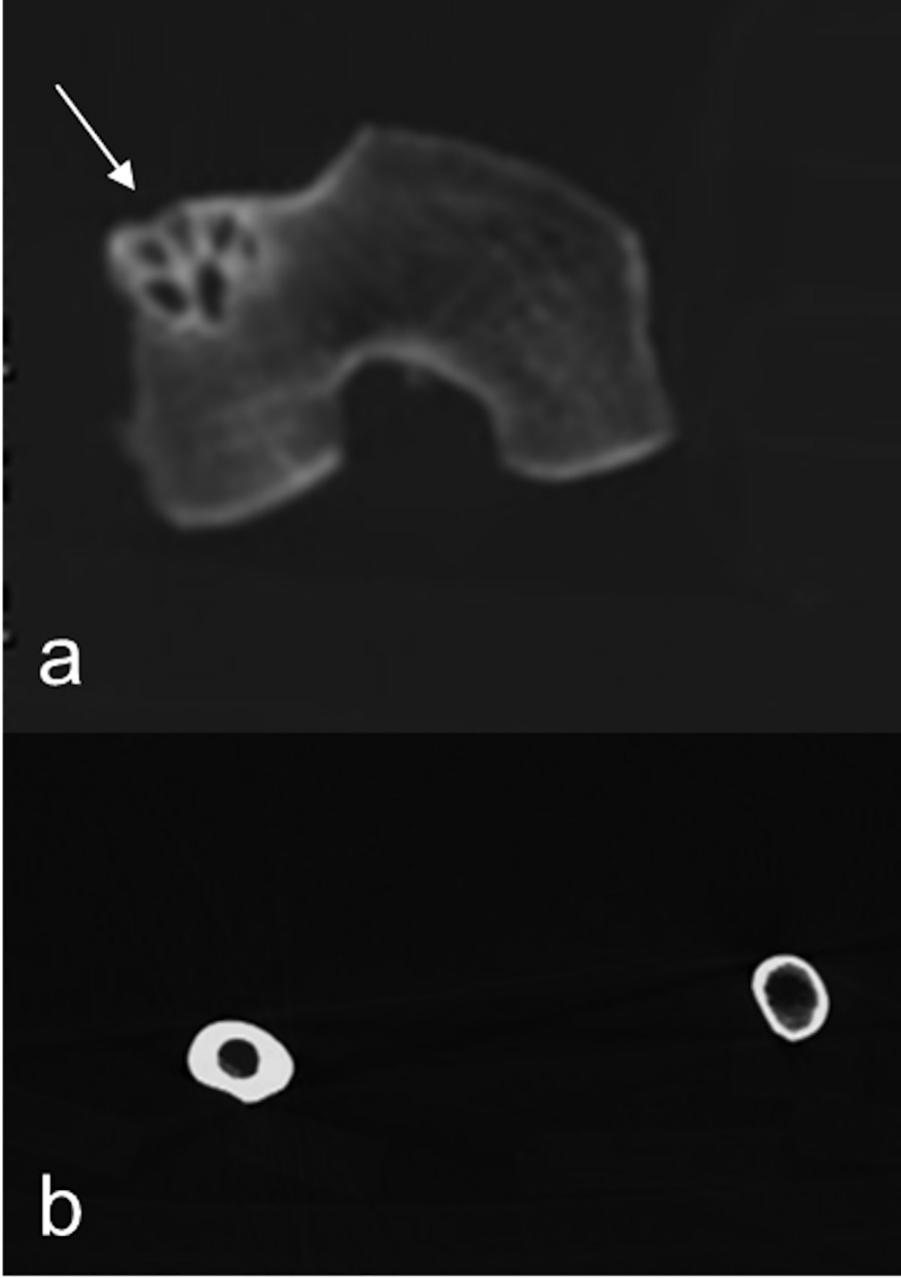
Cystic multiloculated lesion on the lateral condyle of right femur likely related to benign fibrous hystiocytoma (a) associated to contralateral hyperostosis likely due to overload (b).

Subject IND 49 showed a polycystic lesion on the external condyle of the left femur. A contralateral hyperostosis was observed on the right femoral diaphysis (Fig 6).

**Fig 7:**
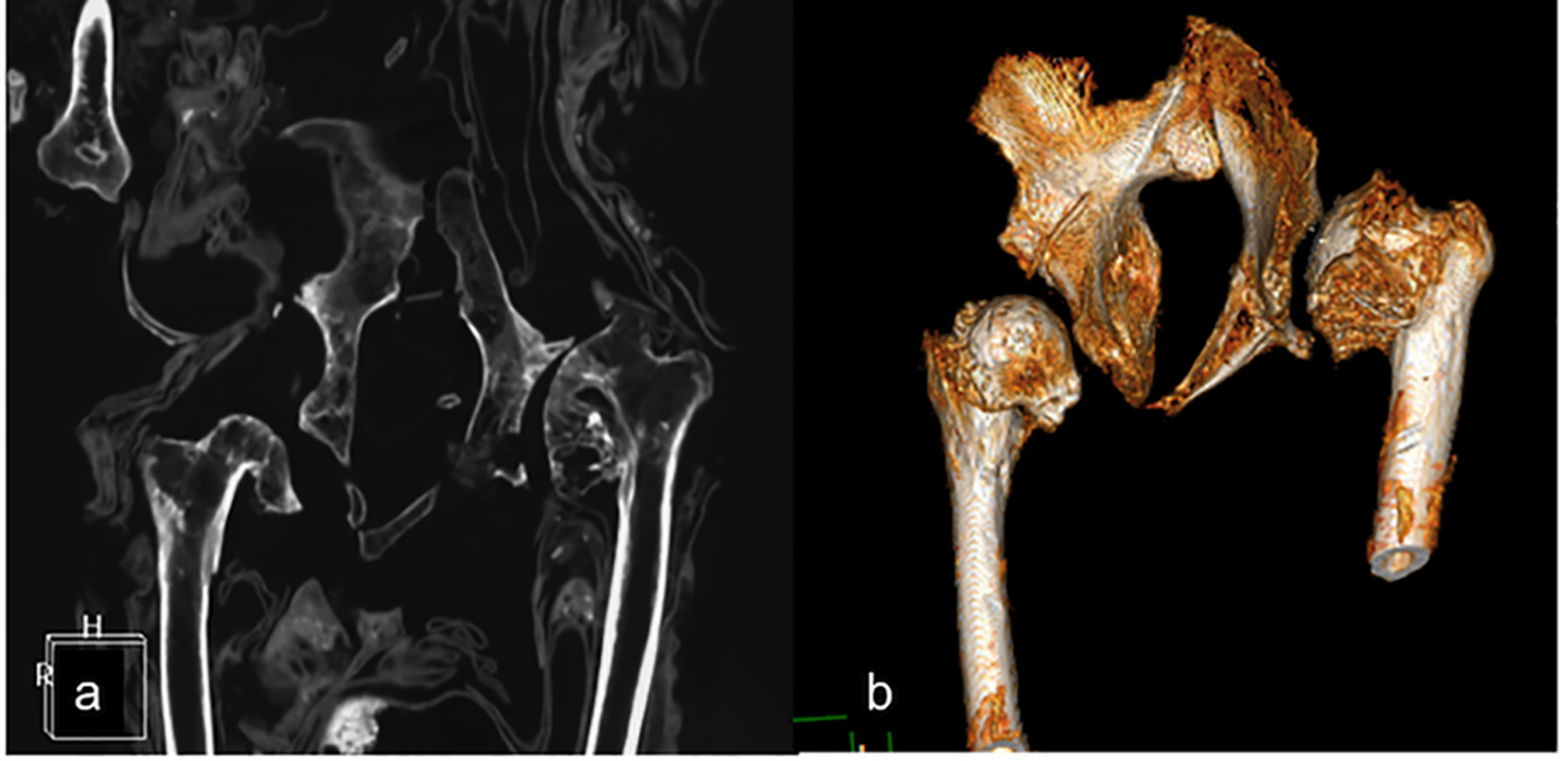
Severe deformation of femoral head related to dysplasia as shown in MPR coronal (a) and VR reconstructions (b).

One individual (IND 12) showed bilateral congenital hip dysplasia characterized by bilateral shallow hip associated with a marked coxo-femoral arthritis (Fig 7). In this case, moderate bilateral arthritis of both the knees were observed as well.

**Fig 8:**
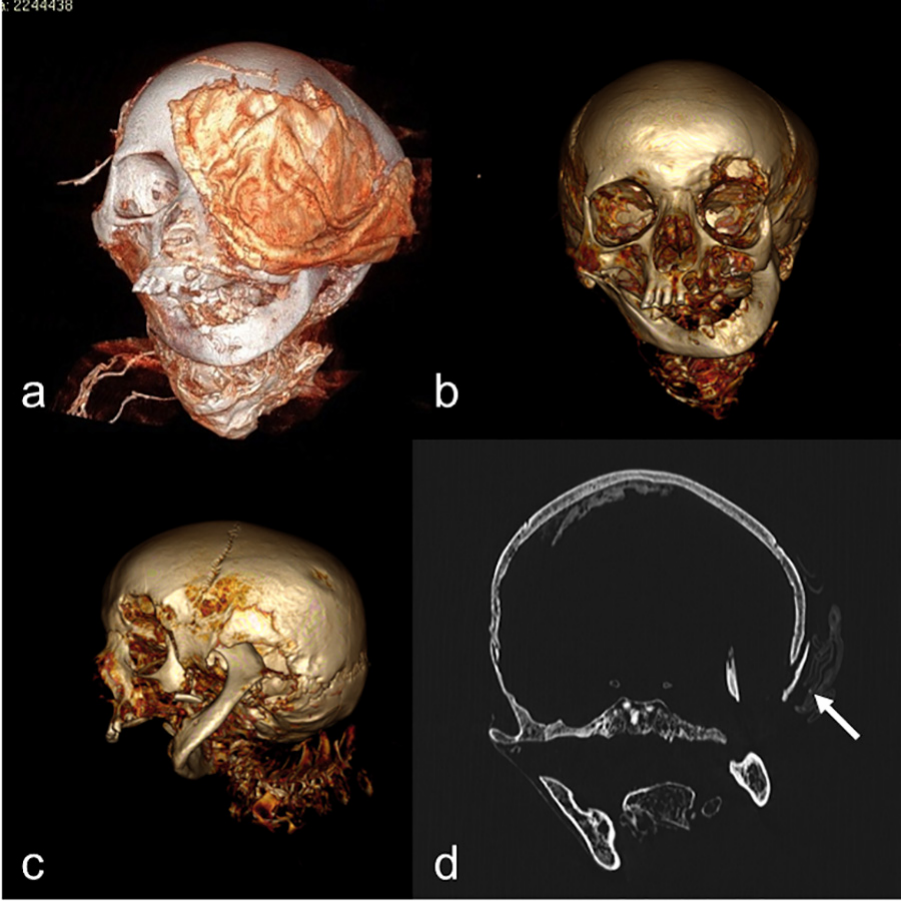
VR reconstructions (a,b,c) and coronal MPR reconstructions (d) of skull. Large bandage on the left orbit to cover a large osteolytic lesion of the left orbit with large erosive phenomenon. The jaw presented an amputation of the left ascending tract at approximately two thirds. A lamellar bony structure was present on the external superior margin of the wide discontinuity of the skull likely due to reparative process (arrow).

A relevant case was represented by the IND 55, the mummy with the only head and cervical spine preserved (Fig 8). Actually the head of IND 55 was affected by a large discontinuity of the basis and of the calvarium on the left temporal region, with a wide destruction of the bone and of the petrous bone. The osteolytic lesion showed irregular and partially sclerotic margins. Moreover the lesion extended to the left orbit with significant bone erosion. The jaw showed an amputation of the left ascending tract at approximately two thirds. The distal margins of the mandible were irregular and sclerotic.

Wide sclerotic changes also involved the omolateral maxillary bone. The lesion extended to the sphenoid which showed a sclerosis on the left side as well. A lamellar bony structure can be seen on the external superior margin of that wide discontinuity of the skull, likely due to a reparative process (Fig 8).

Due to the reduced grading of preservation, analysis of internal organs in our sample of mummies did not add any other information about disease or cause of death.

### Occupational markers

With regards to “daily-activities related”radiological markers, the most common signs observed concerned consequences of the overload such as degenerative changes of distal tibia and fibula, Schmorl hernias and scapolo-humeral arthritis (Table 3) [12].

Even though the mean age of the mummies was relatively high (>50 years old), some of the individuals showed specific changes more related to occupational markers than with aging.

Particularly IND 59 had specific signs of overload on the left upper limbs and IND 57 with signs of overload on the lower limbs. Another case with specific occupational markers was a woman younger than 50 years old (IND 49), who had several Schmorl hernias in the spine.

### Nutritional markers

Some information on nutrition was detectable in our images, particularly each mummy showed a good bone tropism as demonstrated by the lack of Looser lines (nutrition markers).

## Discussion

### CT scan as novel approach to anthropologic investigations

In the past, mummies were only examined by anthropologic and paleo-pathologic methods to reveal anatomic abnormalities, diseases or causes of death. However these methods often caused severe damage or destruction to mummified bodies [3].

**Fig 9:**
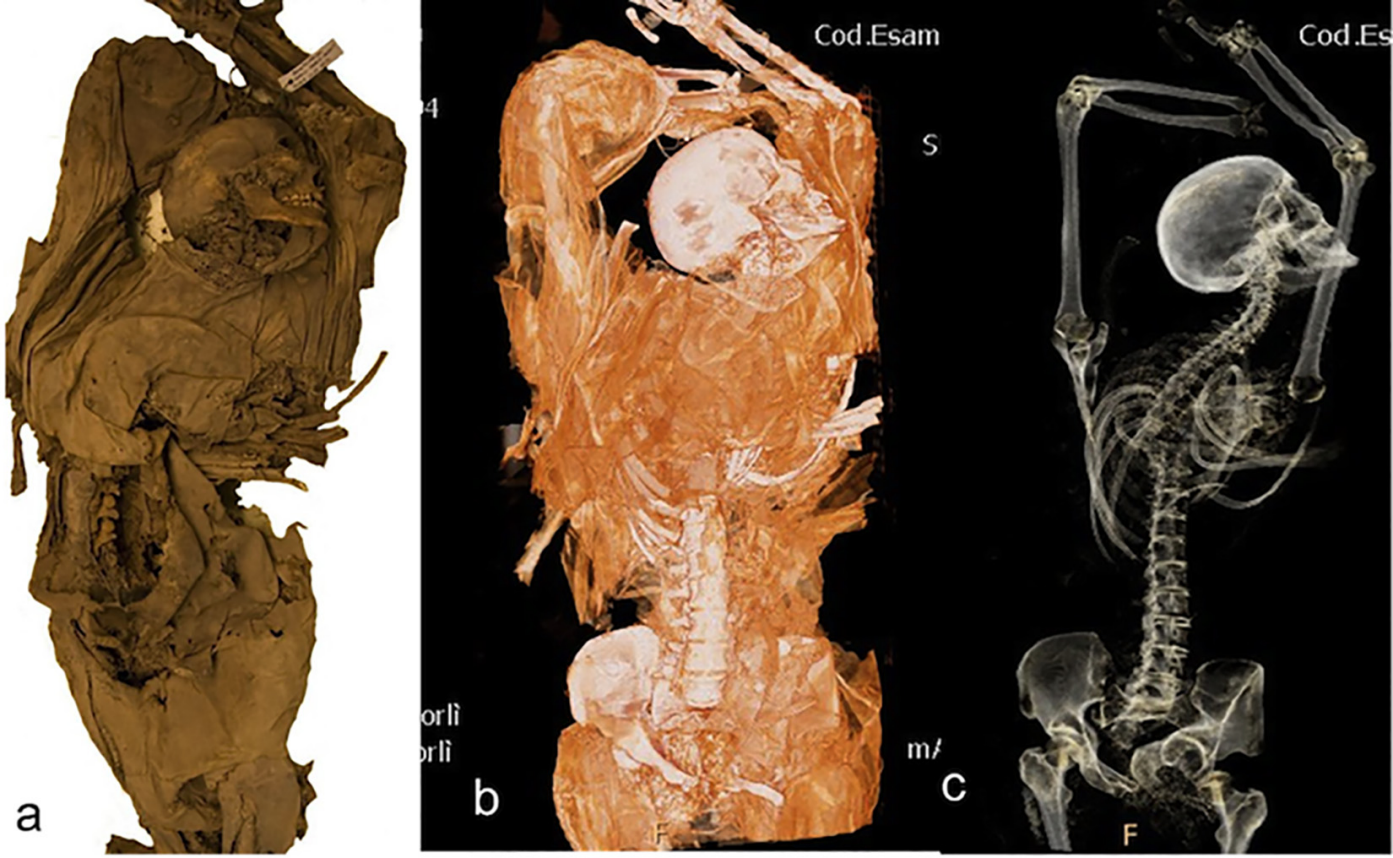
IND 50: female with estimated age of about 20–29 years old. Picture of the mummy (a). VR reconstructions with soft tissue filter of visualization (b) and VR with bone filter of visualization (c).

With the introduction of computed tomography (CT) more detailed imaging was provided for organs, guaranteeing the integrity of the bodies [23–24]. Through the “virtual dissections” of CT scan the wrapping is separated from the other materials and the anatomy is visible (Fig 9) [7].

Most of the reports published in literature concerned Egyptian intentionally mummified dead men and women, few articles can be found on natural mummies such as those in our sample [10].

One of the most relevant clues, is represented by grade of preservation compared with literature. We firstly have adopted the Score System of Panzer et Al [20] in which authors provide a quantitative approach to grade of preservation of mummies.

On the basis of this score, we identified a low score of preservation of soft tissues, particularly for internal organs, in spite of a good integrity of the skeleton.

One of the main reasons for the low grade of preservation derives from the modality of inhumation that consisted in falling from a trap door in the church. Indeed the falling and rolling of new bodies caused ruptures and displacement of bone segments to the underlying bodies.

Another consequence of this modality of inhumation for precipitation consists of the presence of multiple post-mortem fractures (Fig 5B).

Among the reports of CT study of well-preserved natural mummies in the World is the mummy of Similaun Iceman in the Otzal Alps [25–26] and the mummified bodies of Inca children killed as sacrificial victims, discovered in the Llulliaco Mountain in Argentina [27].

Both these above-cited cases presented an exceptional state of preservation due to freezing and dehydration thanks to low temperatures of the altitude.

However these environmental conditions of mummification were very different from those of our population. For this reason, we considered more interesting the comparison with the radiological evaluation of fifteen mummies discovered at Venzone in the region of Friuli, in Italy [10]. Those mummies were analysed either by CT scan or conventional radiology.

Similarly to the mummies from Roccapelago, the mummies from Venzone were buried under the floor of a cathedral.

In the crypt of Venzone, the microclimatic conditions, such as aeration and porous limestone walls and floor, favoured the process of mummification. The mummification process at Venzone provided better results in terms of preservation than in Roccapelago thanks to two elements not identified in our sample: the presence of a fungus (Hypha Bombicina Pers) dehydrating soft tissues and burying of each individual in holes excavated in the wall [10].

As reported in literature, the interior organs of our sample, particularly the digestive system, were completely decomposed and residual soft tissues showed as an amorphous mass without anatomical definition [24]. Among non-bony tissues the only ones recognizable were: tendons, meninges and skin.

### Pathologic findings

With the above limitations, we observed frankly pathological changes only in three mummies (IND 12, 49 and 55).

In one case, the diagnosis was hip dysplasia with high confidence (IND 12). In the remaining two cases (IND 49 and 55) we formulated a differential diagnosis [28].

The IND 49 showed a cystic multiloculated lesion on the meta-epiphysis of the external condyle of the left femur. The absence of bone expansion or of cortical osteolysis oriented our diagnosis to a benign bone lesion. Due to the rarity of not ossifying fibroma in adult age, we considered a diagnosis of benign fibrous histiocytoma with more likelihood (Fig 6A).

Actually this kind of tumour typically affects epiphysis and metaphysis of tubular bones inducing osteolysis, trabeculation and bone sclerosis. Less likely was a diagnosis of giant cell tumour.

In the same individual, a controlateral hyperostosis of femoral diaphysis was observed (Fig 6B). For this finding, we have hypothesized a possible bone thickening secondary to overload due to an antalgic gait.

IND 55 showed a huge osteolytic lesion of the left temporal bone that involved the ipsilateral orbit and caused amputation of the jaw. The huge size of this bone lesion induced us to hypothesize a differential diagnosis between a neoplastic vs infectious lesion. However, evidence of bony tissue with laminar appearance along the upper margin of the lesion oriented to the diagnosis of osteoriparative reaction in osteomyelitis, not to a disruptive neoplastic process. Pathogenesis of this finding could be the consequence of a large infectious oto-mastoiditis with orbital involvement (Fig 8). That pathological change may also have caused the death of the subject. Concerning the other cases we were not able to speculate on cause of death.

### Occupational markers

Examination of degenerative changes of the skeleton allowed us to infer data on daily occupation of this population that were considered “occupational markers” [12].

Seven people showed signs of osteoarthritis with a grading ranging from 1 to 4, and with a mean value of 2.9 according to the Kellegren and Lawrence scale [22].

Actually the people from Roccapelago were devoted to exhausting activities, such as gathering firewood or carpentry, characterized by carrying heavy loads on their shoulders.

Moreover people showed arthritis of the shoulders, chronic tendinopathy of the humerus and severe degenerative changes in the spine.

Several cases of spondyloarthritis including Schmorl hernias, were also observed in young subjects, suggesting heavy loads.

### Nutritional markers

Observing our data, nutritional markers including Looser lines and bone tropism were particularly interesting. Actually the Looser lines were rare and bone tropism was acceptable: these two findings induced us to hypothesize that general conditions of this population were acceptable.

Moreover, anthropological data from the overall population of 281 buried individuals in the crypt confirmed this trend.

### General considerations on the mummified population of Roccapelago

The discovery of the burials in Roccapelago, makes this a unique site in Italy, so much so as to render it a model for comparison for study on regional health during the XVI-XVIII centuries.

Radiological deductions on the limited sample of thirteen mummies was coherent with data coming from the anthropological analysis on the whole mummified population counting about 64 bodies.

Combining this data, we were able to describe the health profile.

Direct physical analysis on 64 more or less mummified individuals, allowed us to reveal that the samples show a good balance between the sexes.

The estimation of the age at death highlighted that the majority of the population (around 50%), both males and females, died when they were over 50, it also highlighted that in mature subjects (30–49 years) there were many more deaths in females compared to males.

The less represented age-band of the mummies was of adolescents and of young adults, with a negative flexion for women. This flexion of the female mummies was probably related to the high risk of labor. At the examination of stature, the mummies show an upward trend of the stature, through the centuries.

Indeed, the average height calculated for SU 23 belonging to XVIII century was almost 168 cm in males and 162 cm in females. On the other hand, the average height recorded in US 28 belonging to XVI and XVII centuries, was about 162.3 cm in males and 150.7 cm in females, with an average increase of 5.5 cm in males and 9.2 cm in females.

This data suggest a relative improvement in living conditions and subsistence for the population between the XVII and XVIII centuries.

With regards to nutrition, two main types of analysis are being carried out on the remains: trace elements and stable isotopes, using the ICP-MS methodology, together with laser ablation. Thanks to the research of the trace elements it has been possible to recognise a predominantly vegetarian diet in the Roccapelago community, linked to the products available on the mountain. In a later period (XVII-XVIII centuries) the diet certainly changed, incorporating limited quantities of meat and cereals [11].

### Epidemiologic data inferred from parish records

**Fig 10:**
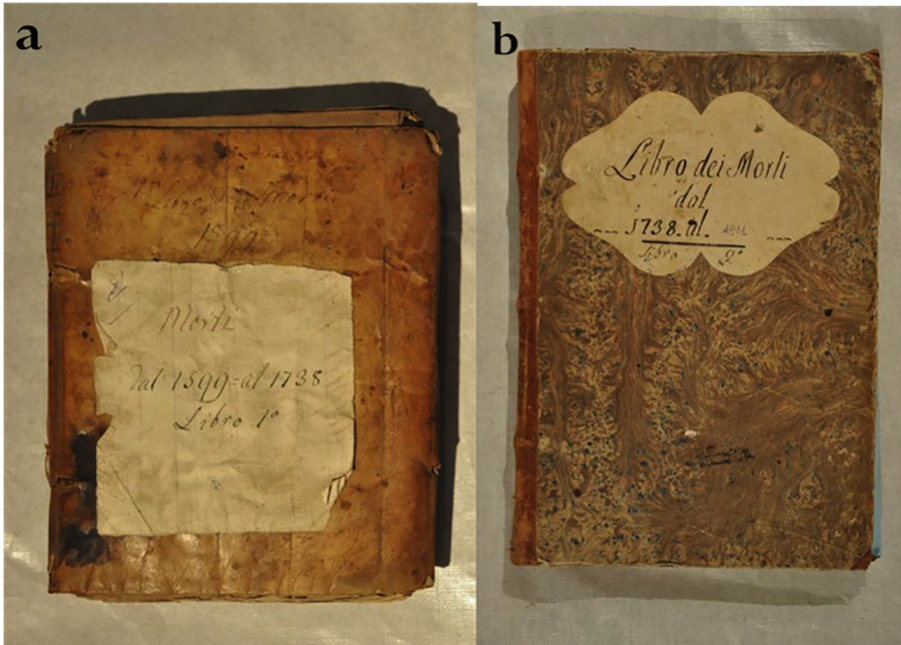
Two ancient parish records: the first from 1599 to 1738 (a), the second one from 1739 to 1891 (b).

**Fig 11:**
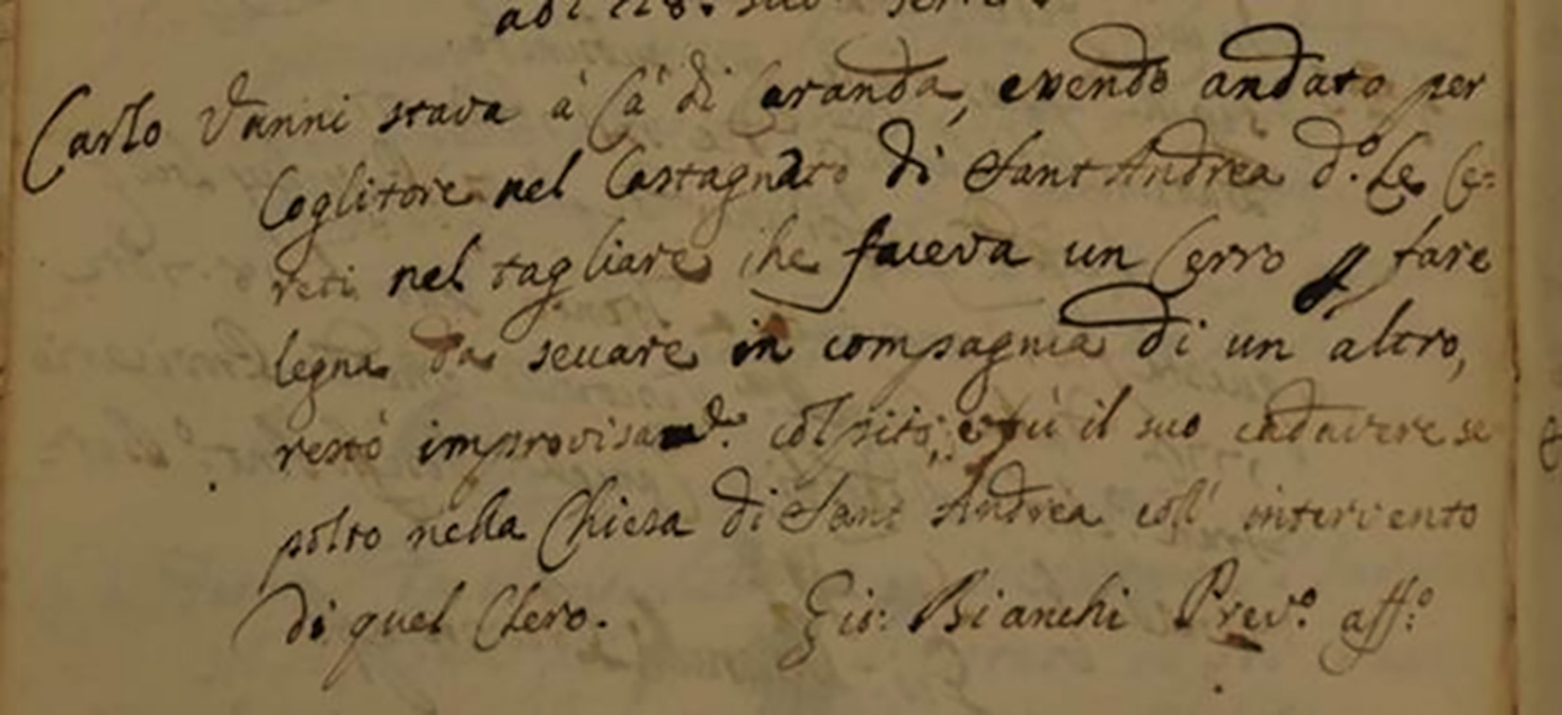
Fragment of the ancient parish records in ancient Italian language: February 28 1773, Carlo Vanni (…) chopping (…) old oak wood making firewood to burn together, he was suddenly struck (the original Italian text: “28 Febbraio 1773 …nel tagliare…un cerro per fare legna da seccare in compagnia …resto improvvisamente colpito) (c).

**Fig 12:**
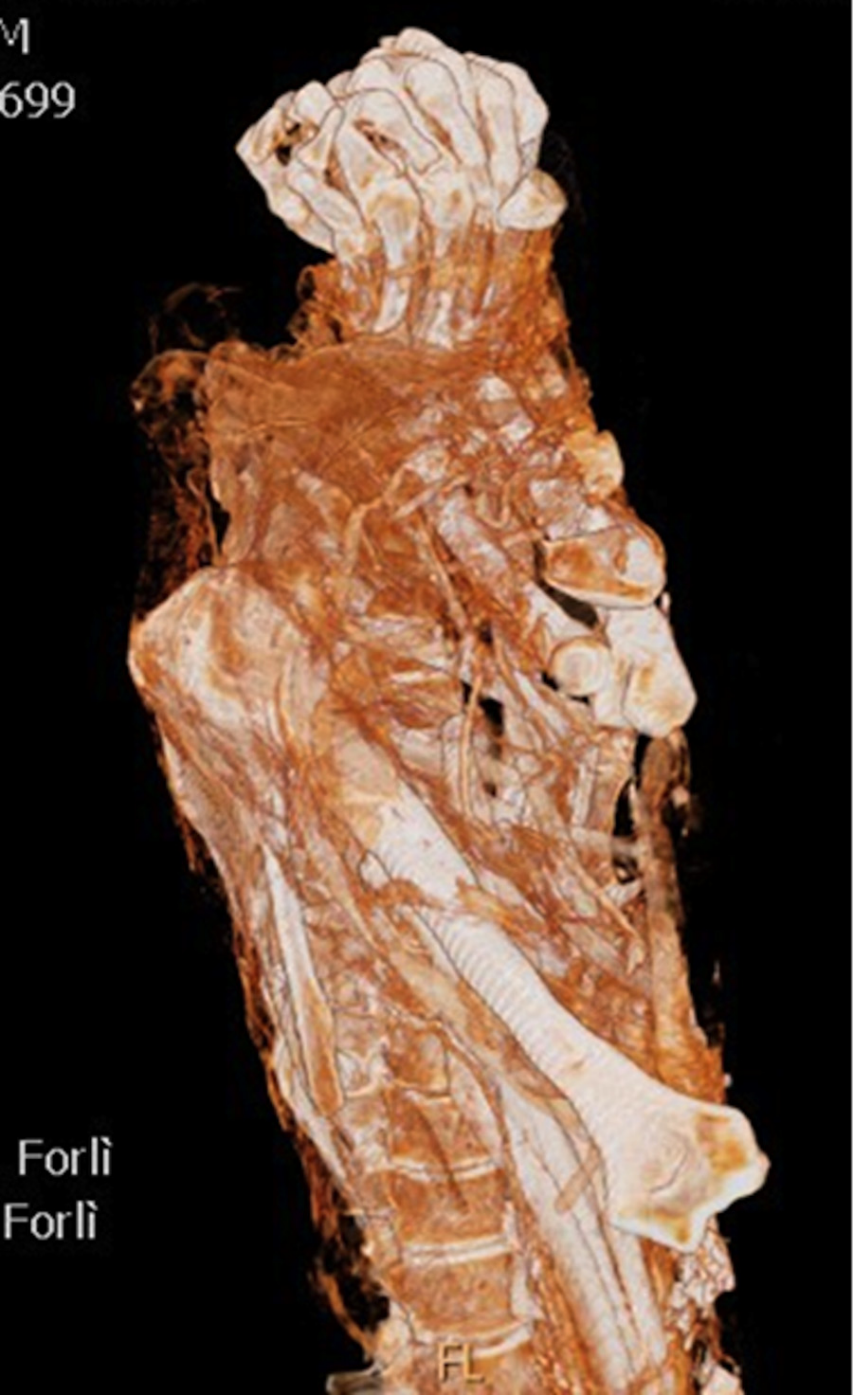
IND 57: VR reconstructions show hands in praying position.

Most of the data concerning mortality of the whole Roccapelago population, comes from the parish records (so called “Records of Deaths”) (Figs 10 and 11). The systematic annotation about causes of death is a relatively late phenomenon in Roccapelago. Rare sporadic records are dating at the end of 1600. Over about 1937 records on causes of death, only the 41% (798 annotations) describes causes of death (Fig 12). The clinical description that were reported in the parish records were classified in sections later. The main sections were based on the prevalence of diseases and included Apoplexy, Cardiac fever, Hidropisy, Illness, pulmonary and worms [28]. Traditionally, these terms had a specific meaning. For example a*poplexy* referred to a sudden death that began with a loss of consciousness.

The term “cardiac” was generally referred to this definition “pain and vomit, and subjects suddenly dead” or “chest pain”. In that group the vasovagal reactions and cardiac symptons were the most frequent entities. The term “fever” included several variants and indicated multiple diseases, particularly “malignant fever”, tertiary fever, maremman fever all regarding malaric fever.

The term “ethic fever” indicated pulmonary tubercolosis. On the other hand, the term “putrid fever” regarded diseases related to gangrene. The term “worms” was often used in reports describing infants’ death and it was usually related to gastrointestinal complications of infections. Records concerning oncological disease were rare. Only three examples were found consisting in dialogues between the doctor and the parish who recorded the cause of death (“disease occurring on the breast with a dilatation below the shoulder the following year”; tumor of the liver; fungus-shaped tumor at the basis of the neck).

### Comparison with contemporary populations

Similar discoveries, even if less numerous, can be recognised in the mummies from Monsampolo del Tronto (Ascoli Piceno). That mummies showed a slightly higher economic wellbeing compared to Roccapelago [29].

However they also show a lower life expectancy, with an expectation of life of 50 years. Whereas in Roccapelago, we know, thanks to the parish register, that it was not unusual living over 70 years [30].

The good life expectancy in Roccapelago was also better if compared with data reported in the rest of Europe, such as for the English people in the late 16th and early 17th centuries, in which it was just below 40–39.7 years [31].

### Non-clinical details and conclusions

**Fig 13:**
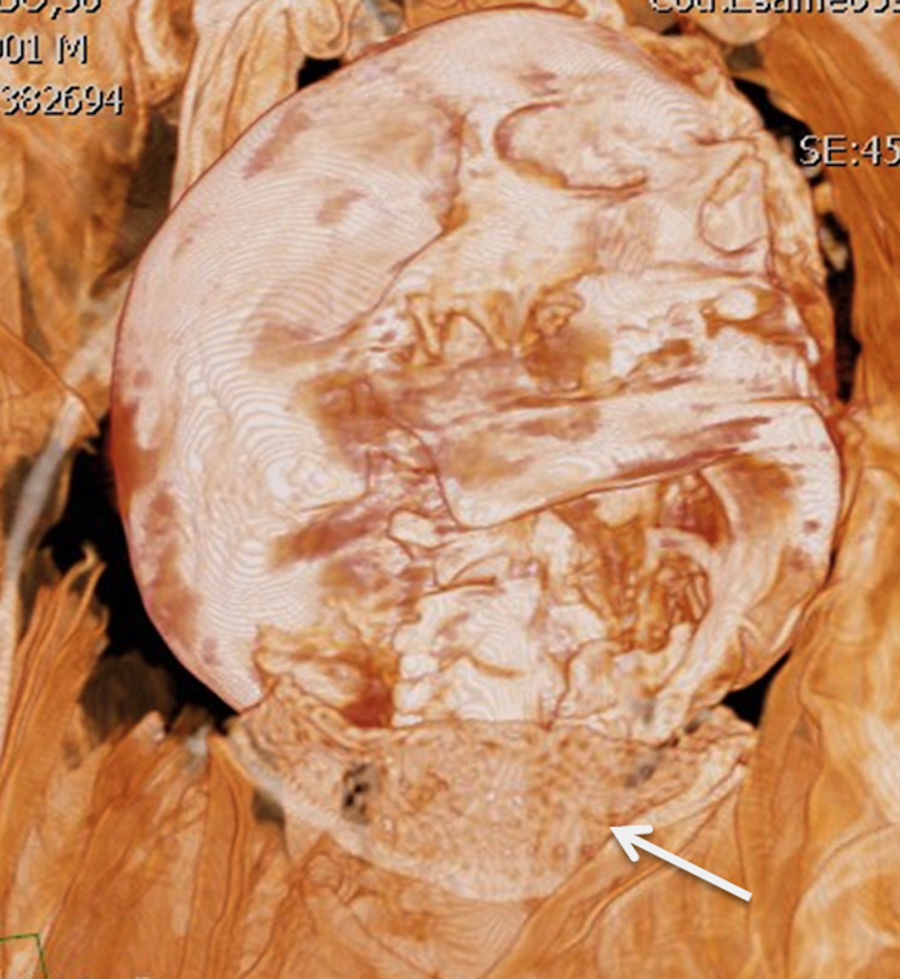
IND 50: Lace on the neck of the blouse (arrow).

Finally some “non-clinical details” of our population intrigued us. They consisted of some attitudes, like the case of a body with hands joined as if praying, testifying devotion and care during the act of burying (Fig 13).

Another detail concerned sepulchral clothes that were sometimes adorned with laces and rich draperies testifying to a relative economic well-being of some individuals.This data has been confirmed by anthropological investigations regarding funerary trousseau consisting of clothes, crosses, medals, rings (including wedding rings), pins, coins, combs and letters. These anthropological and radiological evaluations induce us to consider the population of Roccapelago poor, but not desolate.

Our report has several limitations. First, it analyses a restricted sample of individuals from the community, obtaining little pathological data.

Moreover, in the cases of pathological changes, we can make hypotheses or a differential diagnosis, but we do not have the histological confirmation.

The greatest advance in our report compared with prior publications in literature on mummies, is based on the feature of our population. Actually, the sample does not consist of a close group of individuals with similar and homogenous features (like monks) but of a whole mountain community with heterogeneous characteristics.

The multidisciplinary approach involving anthropologists and radiologists, allowed us to improve knowledge on health conditions and cause of death in these ancient people.
